# A Hematological-Related Prognostic Scoring System for Patients With Newly Diagnosed Glioblastoma

**DOI:** 10.3389/fonc.2020.591352

**Published:** 2020-12-10

**Authors:** Chao Zhao, Long-Qing Li, Feng-Dong Yang, Ruo-Lun Wei, Min-Kai Wang, Di-Xiang Song, Xiao-Yue Guo, Wei Du, Xin-Ting Wei

**Affiliations:** ^1^ Department of Neurosurgery, The First Affiliated Hospital of Zhengzhou University, Zhengzhou, China; ^2^ Department of Orthopedic Surgery, The First Affiliated Hospital of Zhengzhou University, Zhengzhou, China

**Keywords:** glioblastoma, hematological marker, prognostic scoring system, prognosis, inflammation, nutrition, coagulation

## Abstract

**Background:**

Glioblastoma is the most common primary malignant brain tumor. Recent studies have shown that hematological biomarkers have become a powerful tool for predicting the prognosis of patients with cancer. However, most studies have only investigated the prognostic value of unilateral hematological markers. Therefore, we aimed to establish a comprehensive prognostic scoring system containing hematological markers to improve the prognostic prediction in patients with glioblastoma.

**Patients and Methods:**

A total of 326 patients with glioblastoma were randomly divided into a training set and external validation set to develop and validate a hematological-related prognostic scoring system (HRPSS). The least absolute shrinkage and selection operator Cox proportional hazards regression analysis was used to determine the optimal covariates that constructed the scoring system. Furthermore, a quantitative survival-predicting nomogram was constructed based on the hematological risk score (HRS) derived from the HRPSS. The results of the nomogram were validated using bootstrap resampling and the external validation set. Finally, we further explored the relationship between the HRS and clinical prognostic factors.

**Results:**

The optimal cutoff value for the HRS was 0.839. The patients were successfully classified into different prognostic groups based on their HRSs (P < 0.001). The areas under the curve (AUCs) of the HRS were 0.67, 0.73, and 0.78 at 0.5, 1, and 2 years, respectively. Additionally, the 0.5-, 1-y, and 2-y AUCs of the HRS were 0.51, 0.70, and 0.79, respectively, which validated the robust prognostic performance of the HRS in the external validation set. Based on both univariate and multivariate analyses, the HRS possessed a strong ability to predict overall survival in both the training set and validation set. The nomogram based on the HRS displayed good discrimination with a C-index of 0.81 and good calibration. In the validation cohort, a high C-index value of 0.82 could still be achieved. In all the data, the HRS showed specific correlations with age, first presenting symptoms, isocitrate dehydrogenase mutation status and tumor location, and successfully stratified them into different risk subgroups.

**Conclusions:**

The HRPSS is a powerful tool for accurate prognostic prediction in patients with newly diagnosed glioblastoma.

## Introduction

Glioblastoma multiforme (GBM) is the most common and lethal type of cerebral tumor, accounting for 15.1% of all central nervous system tumors, with an incidence rate of 3.19 per 100,000 individuals (1, 2). Currently, the standard treatment for these patients consists of maximally safe surgical resection, followed by concurrent chemoradiotherapy and adjuvant therapy with temozolomide (TMZ). Even after standard intervention, patients with GBM only have a median overall survival (OS) of approximately 15 months and only a small proportion of them, approximately 5%, survive at 5 years (3). It is known that the important prognostic factors of GBM include age; performance status; extent of resection; treatment; and various molecular markers, i.e., epidermal growth factor receptor, methylation status of the gene promoter for O6-methylguanine-DNA methyltransferase (*MGMT*), and isocitrate dehydrogenase (IDH)-1/2 mutations (2–8). The preoperative detection of molecular markers is invasive and technologically demanding due to the unique blood–brain barrier (9); therefore, there is an urgent need for more readily accessible predictive factors that are detected *via* noninvasive procedures and are more cost-effective to develop individualized treatments for patients with newly diagnosed GBM.

Mounting evidence has revealed that preoperative hematological biomarkers, which reflect the tumor microenvironment of the body to a certain extent, could serve as diagnostic and prognostic markers for human cancers (10–12). Previous investigations have indicated that markers of inflammatory response, such as the neutrophil-to-lymphocyte ratio (NLR), platelet-to-lymphocyte ratio (PLR), and monocyte-to-lymphocyte ratio (MLR), are associated with the clinical outcomes of gliomas, especially GBM (13–15). Several studies have indicated that a state of preoperative hypercoagulability is related to poor prognosis in patients with GBM (16, 17). Other factors such as glucose (GLC) (18, 19), hemoglobin (HBG) (20, 21), the prognostic nutrition index (PNI) (14, 22, 23), lactate dehydrogenase (LDH) (24, 25) and red blood cell distribution width (RDW) (20, 26) have also been shown to have prognostic value in GBM. Fortunately, these hematological indicators can be obtained from convenient and inexpensive preoperative clinical routine tests. However, the hematological prognostic indicators reported in previously published papers only reflect a single aspect of the body (13, 16, 23), which is insufficient due to the complexity of the human internal environment. A comprehensive scoring system would make it possible for a single index to reflect inflammation, nutrition, and coagulation statuses simultaneously.

Thus, in the present study, we collected all hematological indicators with proven prognostic significance and adjusted these indicators to binary variables based on the analysis of receiver-operating characteristic (ROC) curves. Subsequently, we developed a hematological-related prognostic scoring system (HRPSS) using the least absolute shrinkage and selection operator (LASSO) Cox proportional hazards regression analysis for the first time (27), which is an appropriate solution to establish signatures if there are numerous correlated covariates. We then comprehensively evaluated the predictive ability of the HRPSS and constructed a nomogram to quantitatively predict patients’ survival. Finally, we further investigated the association between the hematological risk score (HRS) and clinical prognostic factors to explore a novel preoperative risk stratification system for GBM.

## Materials and Methods

### Patients

This retrospective study included patients who were newly diagnosed with glioblastoma at the First Affiliated Hospital of Zhengzhou University between June 2016 and January 2019, following the Medical Ethics Committee approval. The inclusion criteria were as follows: 1) patients aged ≥ 21 years old; 2) patients with GBM confirmed by histopathology; 3) patients with data of routine blood test, biochemical, and coagulation results before the surgery; and 4) radiotherapy plus concomitant and adjuvant temozolomide was the Stupp regimen; only adjuvant chemotherapy was the TMZ plan. The exclusion criteria were as follows: 1) patients with obvious infection or autoimmune diseases; 2) patients with hematological diseases; 3) patients with other malignancies; and 4) perioperative surgery-related mortality. Ultimately, 326 patients were included in this study with complete demographic, clinical, and pathological data available. Each patient was followed up regularly until death or June 2020. The patients were observed once every month in the first 6 months after surgery and then every 3–6 months thereafter. Finally, all patients were randomly divided into a training set (n=228, 70%) and external validation set (n=98, 30%) using a random seed set in 2020.

### Data Collection

The following variables were obtained for each patient: age at diagnosis; sex; first presenting symptoms; mean clinical history; preoperative Eastern Cooperative Oncology Group performance status (ECOG PS) score; tumor size; tumor location; extent of resection; IDH mutation status; M*GMT* methylation status; adjuvant therapy; and laboratory index values, such as neutrophil counts, lymphocyte counts, platelet counts, monocyte counts, HBG levels, LDH levels, fibrinogen (FIB) levels, D-dimer (DD) levels, RDW, and serum levels of GLC and albumin, which were collected from our hospital case documents. The hematological data were all collected in the routine preoperative blood test before any treatment, including radiotherapy, chemotherapy, or surgery. Routine blood, coagulation function and hepatic function tests were centrally performed at the Department of Clinical Laboratory within 2 h of blood sample collection. The NLR, PLR, and MLR were defined as the ratios of absolute neutrophil counts, platelet counts, and monocyte counts divided by the absolute lymphocyte counts, respectively. The PNI was calculated using the serum albumin value (g/L) + 0.005 × peripheral lymphocyte count/mm^3^ (28). OS was calculated from the date of tumor resection to the date of last follow-up or death. Mean clinical history referred to the time from onset of symptoms to hospital admission.

### Hematological Data Processing

In the overall database, the optimal cutoff value was calculated for each hematological index based on analysis of the ROC curve. In the training set, the hematological indices were adjusted to binary variables according to the cutoff values to improve the generalization of the data. Specifically, when a certain index value for a patient was higher than the cutoff value, the score of this index was 1; otherwise, it was 0. The validation set used the same cutoff values for the same data processing.

### Development and Validation of the HRPSS

First, univariate Cox regression analysis was used to screen out prognosis-related indices using the data of the training set (n=228). The LASSO Cox regression analysis was performed on the aforementioned hematological biomarkers to determine the optimal model composed of 9 hematological markers. Subsequently, the HRS was calculated for each patient. ROC curve analysis was used to determine the optimal cutoff value of the HRS, which divided patients into high-risk and low-risk groups. Survival rates were calculated using the Kaplan–Meier (K-M) method, and the significance of differences between the survival curves was determined using the log-rank test. A time-dependent ROC curve was generated to evaluate the diagnostic performance of the HRS in terms of survival time. The areas under the curves (AUCs) corresponding to 0.5, 1, and 2 years were calculated to measure the prognostic ability. In addition, the prognostic predictive power of the HRPSS was further validated in the external validation set (n=98) using the same cutoff value. Finally, the HRS was demonstrated as an independent prognostic factor based on univariate and multivariate Cox regression analyses in both datasets.

### Construction and Evaluation of the Nomogram

First, all clinical covariates were included in the univariate Cox regression analysis before module construction in the training group. Multivariate Cox analysis was then performed with all significant (P < 0.05) covariates including the age, ECOG PS score, first presenting symptoms, tumor location, surgical resection, therapy status, IDH mutation status, and HRS. The hazard ratio and P-value of each covariate of the Cox analysis were shown by a forest plot. A nomogram was formulated based on the results of the multivariate Cox regression analysis. The prognostic performances of factors used to construct the nomogram were assessed using AUCs obtained from the time-dependent ROC curves. The C-index was used to evaluate the discriminative ability of the nomogram, and a relevant calibration plot was generated to assess the accuracy of the nomogram. Decision curve analysis (DCA) was used to evaluate the clinical application of the nomogram. During external validation of the nomogram, the total points of each patient in the validation group were calculated according to the established nomogram, and Cox regression was then performed in this group using the total points as a factor, and the C-index and calibration curve were eventually obtained based on the regression analysis.

### Exploration of the Relationship Between the HRS and Clinical Characteristics

In all 326 patients, the relationship between the HRS and traditional clinical features, such as age, first presenting symptoms, IDH mutation status, and tumor location, was further researched. The prognostic ability of the HRS was then explored among different subgroups, which were divided by all the significant clinical features. Considering the particularity of the IDH mutant population, we performed further subgroup analysis of the IDH mutant and wild-type populations respectively. In addition, the patients were divided into four groups according to the HRS (low- or high-risk groups) and IDH mutation status (mutant or wild-type groups), and the differences in survival time were evaluated among the four groups. The same analysis was also carried out using the other two prognostic factors: age and first presenting symptoms.

Considering the important prognostic significance of MGMT methylation status, we conducted a separate analysis of standard chemotherapy patients who had MGMT methylation analysis. Based on the results of multiple Cox regression analysis of the population, a nomogram including MGMT status, HRS and other clinical characteristics was developed

### Statistical Analysis

Descriptive statistics were used to assess any differences between datasets using the t-test or Mann–Whitney U test for continuous variables and the chi-square test or Kruskal–Wallis test for categorical variables. All statistical analyses were conducted using SPSS software, version 21 (IBM Corp., Chicago, IL), and R software, version 3.3.0 (Institute for Statistics and Mathematics, Vienna, Austria). P values < 0.05 were considered to indicate statistical significance.

## Results

### Patient Characteristics

A total of 392 patients with GBM were identified from our database, and 326 patients were finally enrolled. Of them, 228 and 98 patients with GBM were randomly divided into the training set and external validation set, respectively. The demographics and clinical characteristics of patients in the training set and patients in the validation set are summarized in **Table 1**. In general, there were no significant differences between the training and validation datasets (all P > 0.05). All patients underwent surgery with examinations of IDH-1/2 mutations. However, only 69 standard chemotherapy patients underwent postoperative MGMT methylation analysis. GBM tended to occur in middle-aged men. The mean ages of patients were 54.6 (range, 21 to 80) years and 55.1 (range, 21 to 85) years in the training set and validation set, respectively. There were 134 (58.8%) men and 94 (41.2%) women in the training set and 56 (57.1%) men and 42 (42.9%) women in the validation set. During the follow-up process, the mean OS durations were 14.3 (range, 2 to 41) and 14.0 (range, 2 to 39) months for patients in the training set and validation set, respectively. The optimal cutoff values of the 10 hematological indices in the training set, including the GLC level, HBG level, LDH level, FIB level, DD level, RDW, NLR, PLR, MLR, and PNI, are displayed in **Supplementary Table S1**. The clinical characteristics of 69 patients undergoing MGMT methylation analysis are showed in **Supplementary Table S2**. The mean OS of MGMT methylated patients were 19.4 months, while the mean OS of MGMT unmethylated patients were 14.4 months.

**Table 1:** Summary of clinical characteristics of GBM patients.

Characteristic	Training Group (n = 228, 70%)	Validation Group (n = 98, 30%)	P-Value
**Sex**			
Male	134(58.8%)	56(57.1%)	0.784
Female	94(41.2%)	42(42.9%)	
**Age, years**			
Mean	54.6	55.1	0.734
Range	21–80	21*–*85	
**First Presenting Symptom**			
Seizures	45(19.7%)	17(17.3%)	0.614
Others	183(80.3%)	81(82.7%)	
**Clinical History, days**			
Median	20.5	20	0.395
Range	1*–*1825	1*–*1075	
**ECOG PS**			
0 1 2	75(32.9%) 129(56.6%) 21(9.2%)	25(25.5%) 63(64.3%) 9(9.2%)	0.574
3	3(1.3%)	1(1.0%)	
**Size, cm**			
<=5	134(70.2%)	57(69.6%)	0.919
>5	94(29.8%)	41(30.4%)	
**Surgery Resection**			
Gross Total	205(89.9%)	86(87.8%)	0.564
Partial	23(10.1%)	12(12.2%)	
**Tumor Location**			
Frontal	50(21.9%)	20(20.4%)	0.788
Temporal	40(17.5%)	19(19.4%)	
Mixed	85(37.3%)	41(41.8%)	
Thalamus	9(3.9%)	2(2.0%)	
Others	44(19.3%)	16(16.3%)	
**Therapy Status**			
Chemoradiotherapy	155(68.0%)	56(57.1%)	0.738
Chemotherapy	39(17.1%)	31(31.6%)	
None	34(14.9%)	11(11.2%)	
**IDH**			
Mutant	37(16.2%)	14(14.3%)	0.658
Wildtype	191(83.8%)	84(85.7%)	
**RDW, %**			
<=12.8	75(32.9%)	24(24.5%)	0.130
>12.8	153(67.1%)	74(75.5%)	
**HBG, g/L**			
<=114	26(11.4%)	11(11.2%)	0.963
>114	202(88.6%)	87(88.8%)	
**G, mmol/L**			
<=5.4	157(68.9%)	66(67.3%)	0.788
>5.4	71(31.1%)	32(32.7%)	
**LDH, U/L**			
<=179	118(51.8%)	55(56.1%)	0.469
>179	110(48.2%)	43(43.9%)	
**FIB, g/L**			
<= 3.3	147(64.5%)	64(65.3%)	0.885
>3.3	81(35.5%)	34(34.7%)	
**DD, mg/L**			
<=0.15	148(64.9%)	65(66.3%)	0.806
>0.15	80(35.1%)	33(33.7%)	
**NLR**			
<=2.3	120(53.6%)	41(58.2%)	0.096
>2.3	108(47.4%)	57(41.8%)	
**PLR**			
<=97.7	69(30.3%)	34(34.7%)	0.430
>97.7	159(69.7%)	64(65.3%)	
**MLR**			
<=0.59	79(34.6%)	45(45.9%)	0.072
>0.59	149(65.4%)	53(54.1%)	
**PNI**			
<=54.8	181(79.4%)	77(78.6%)	0.868
>54.8	47(20.6%)	21(21.4%)	
**OS, months**			
Mean	14.3	14.0	0.769
Range	2*–*41	2*–*39	
**Survival Status**			
Alive	51(22.4%)	18(18.4%)	0.462
Dead	177(77.6%)	80(81.6%)	

ECOG PS, the Eastern Cooperative Oncology Group performance status score; IDH, isocitrate dehydrogenase-1/2 mutations; RDW, red blood cell distribution width; HBG, hemoglobin; GLC, glucose; LDH, lactate dehydrogenase; FIB, fibrinogen; DD, D-dimer; NLR, the neutrophil-to-lymphocyte ratio; PLR, the platelet-to-lymphocyte ratio; MLR, the monocyte-to-lymphocyte ratio; PNI, the prognostic nutrition index; OS, overall survival; Chemoradiotherapy: radiotherapy plus concomitant and adjuvant temozolomide; Chemotherapy: only adjuvant chemotherapy with temozolomide; None: without any postoperative adjuvant treatment.

### Definition of the HRPSS in the Training Group

**Figure 1:**
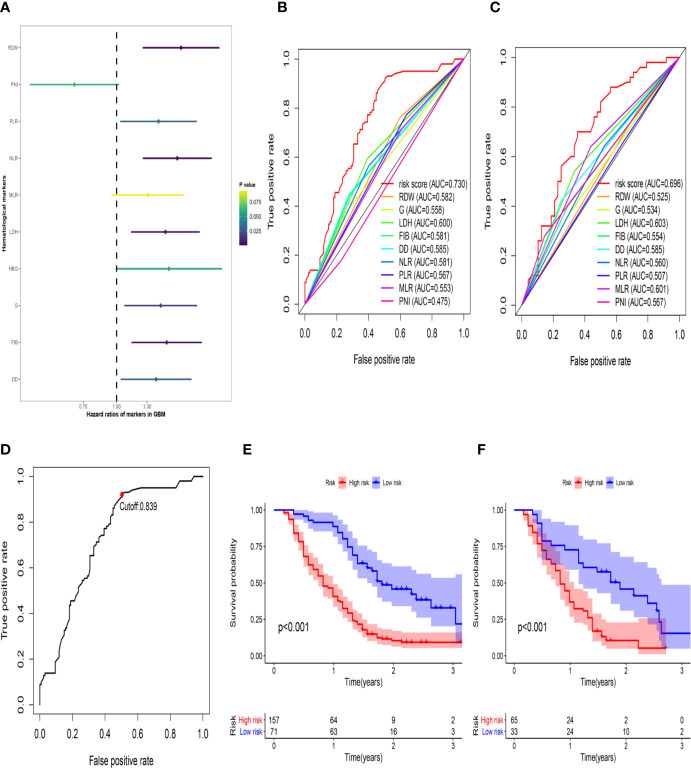
The development of the HRS. **(A)** Univariate Cox regression analysis of the initially hematological markers. The color of the horizontal lines represents the correlation P-value. **(B)** The build results of the HBRPSS were shown in the form of ROC curves at 1-yr in the training group; **(C)** The verification results of the HBRPSS were shown in the form of ROC curves at 1-yr in the validation group; **(D)** The optimal cut-off value of HRS calculated by the analysis of ROC curve; **(E)** Kaplan–Meier curves of overall survival according to HRS-risk groups in the training group; **(F)** Kaplan–Meier curves of overall survival according to HRS-risk groups in the external validation group.

**Figure 2:**
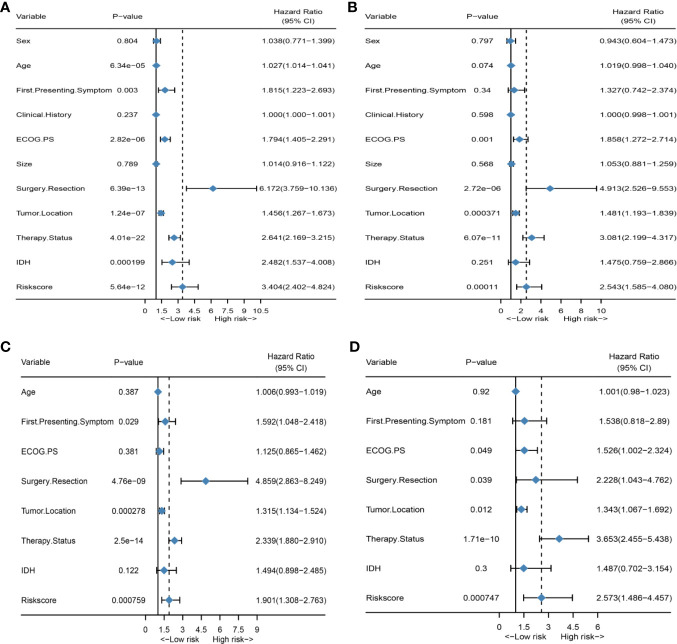
HRS is an independent prognosis factor in both the training set and external validation set. **(A)** Forest plot of univariate Cox regression analysis of all clinical covariates in the training group; **(B)** Forest plot of univariate Cox regression analysis of all clinical covariates in the external validation group; **(C)** Forest plot of multivariate Cox regression analysis of all significant clinical covariates in the training group; **(D)** Forest plot of multivariate Cox regression analysis of all significant clinical covariates in the external validation group.

**Figure 3:**
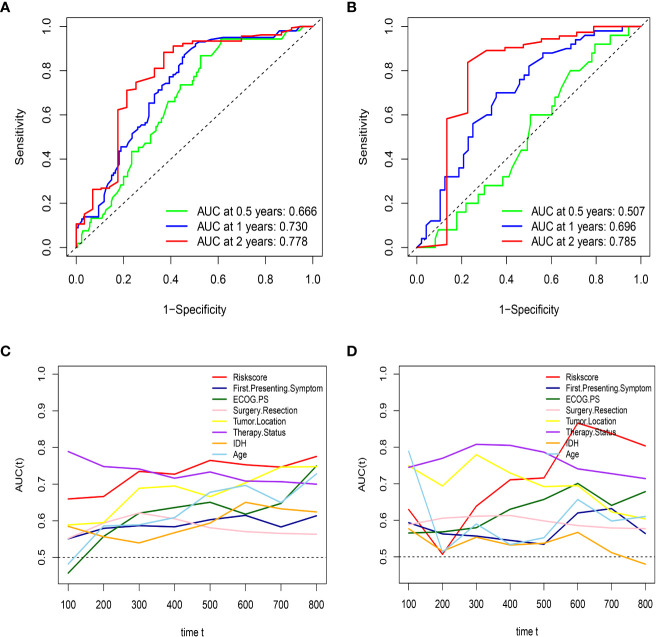
HRS has excellent predictive ability in both the training set and external validation set. **(A)** the AUCs of HRS at 0.5, 1, and 2 years in the training group; **(B)** the AUCs of HRS at 0.5, 1, and 2 years in the external validation; **(C)** Time-dependent ROC curves of the HRS and important clinical characteristics in the training group; **(D)** Time-dependent ROC curves of the HRS and important clinical characteristics in the external validation group.

First, to investigate the relationship between hematological indices and OS, univariate Cox regression analysis was performed in the training group. As shown in **Figure 1A**, since seven of the 10 hematological indices with P < 0.05 and all the indices with P < 0.1 were considered significant. Thus, we included them in the screening of the HRS. Second, we utilized LASSO Cox regression analysis in these indices to establish an HRPSS, finally selecting nine indices that appeared as stable factors. The optimal coefficients used in the calculation formula of the HRPSS are shown in **Supplementary Table S1**. Ultimately, the HRPSS was established based on a linear combination of the selected prognostic indices weighted by their coefficients. The build results of the HRPSS are shown in the form of ROC curves, revealing that the AUC at 1 year of the HRS was larger than any single index used to build it (0.73 vs. 0.48–0.60; **Figure 1B**). As shown in **Figure 1D**, the optimal cutoff value of the HRS was 0.839, which classified patients with GBM into low-risk and high-risk groups. Compared with the OS of patients in the low-risk group, the OS of patients in the high-risk group was significantly poorer (P < 0.001; **Figure 1E**). Subsequently, we conducted univariate and multivariate Cox regression analyses to explore whether the HRS can be used as an independent predictor of prognosis. As shown in **Figures 2A** and **C**, after adjusting for variables such as the age, ECOG PS score, first presenting symptoms, tumor location, surgical resection, therapy status, and IDH mutation status, the results showed that the HRS was an independent prognostic factor (P < 0.001). In the training group, the predictive performance of the HRS and significant clinical characteristics was assessed using the AUCs obtained from the time-dependent ROC curves. As noted in **Figure 3A**, the AUCs of the HRS were 0.67, 0.73, and 0.78 at 0.5, 1, and 2 years, respectively, which indicated that the risk signature had excellent predictive power. **Figure 3C** showed that the therapy status had the highest predictive ability for short-term survival (OS < 1 year), followed by the HRS, while for long-term survival (OS >1 year) prediction, the HRS showed a relatively higher AUC than that for other clinical characteristics.

### Validation of the HRPSS in the External Validation Group

To further validate the robustness of the HRPSS, the patients were divided into high- and low-risk groups by applying the same formula and HRS cutoff value (0.839) in the external validation set. The verification results of the HRPSS in the validation set were also shown in the form of ROC curves, indicating that the AUC at 1 year of the HRS was larger than any single index used to build it (0.70 vs. 0.51–0.60; **Figure 1C**). As shown in **Figure 1F**, the K-M curve revealed that the patients in the low-risk group showed a longer OS compared with that in the high-risk group (P < 0.001). Additionally, we also demonstrated that the HRS was an independent prognostic factor of GBM using univariate and multivariate Cox regression analyses (P < 0.001; **Figures 2B, D**). As shown in **Figure 3B**, the AUCs of the HRS were 0.51, 0.70, and 0.79 at 0.5, 1, and 2 years, respectively, which demonstrated that the HRS is reliable in different datasets. Similarly, as shown in **Figure 3D**, the excellent predictive performance of the HRS on long-term survival was verified by the time-dependent ROC curve in the validation group.

### Establishment and Assessment of a Nomogram Based on the HRS and Clinical Characteristics

**Figure 4:**
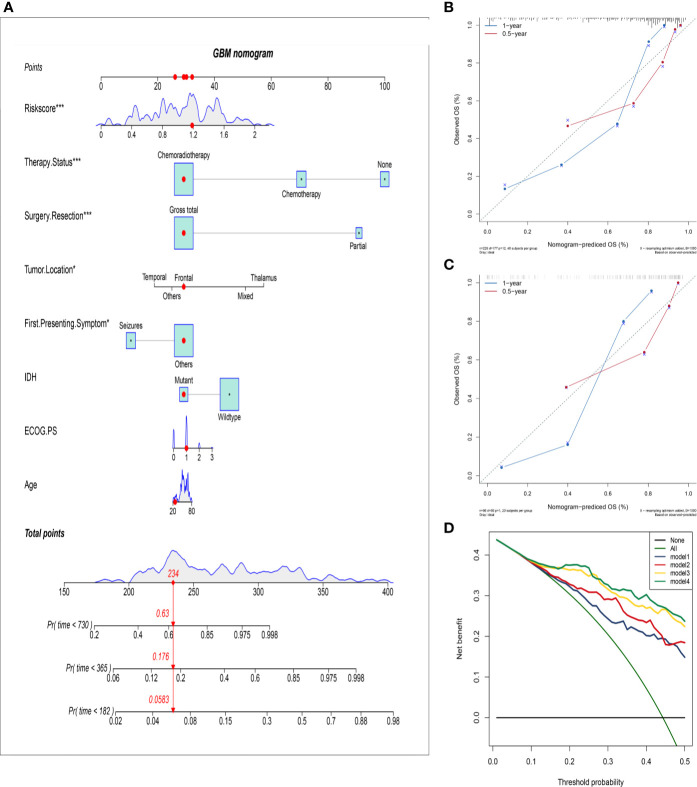
Establish and validate an overall survival nomogram to predict a glioblastoma patient prognosis. **(A)** Nomogram to predict the probability of patient mortality based on HRS and clinical characteristics; **(B)** Calibration chart in the training group to verify the accuracy of the nomogram. Nomogram performance is shown by the plot, relative to the 40-degree line, which represents the ideal prediction; **(C)** Calibration chart in the external validation group to verify the accuracy of the nomogram; **(D)** Decision curve analysis of nomogram in the training group; model 1: preoperative clinical parameters; model 2: preoperative clinical parameters + HRS; model 3: preoperative and postoperative clinical parameters; model 4: preoperative and postoperative clinical parameters + HRS.

To further improve the accuracy of prognosis prediction for GBM, we constructed a nomogram based on the HRS and clinical characteristics in the training group (**Figure 4A**). Each covariate was assigned a score based on Cox proportional hazard ratios, and the nomogram score was the total points obtained by summing the risk point scores of seven covariates. In the nomogram, the HRS had the highest score compared to those of other preoperative factors (ranging from 0 to 60). The C-index for this nomogram was 0.80, and the calibration plot of the proposed nomogram model showed that the predicted 0.5-year and 1-year OS corresponded closely to the actual survival times revealed by K-M analysis (**Figure 4B**). The results of DCA showed that the nomogram could yield clinical net benefits (**Figure 4D**). In the validation cohort, the C-index of the nomogram for predicting OS was 0.82, and a calibration curve showed a relatively good agreement between the prediction and observation in the probability of 0.5- and 1-year survival (**Figure 4C**).

### Association Between the HRS and Clinical Characteristics

**Figure 5:**
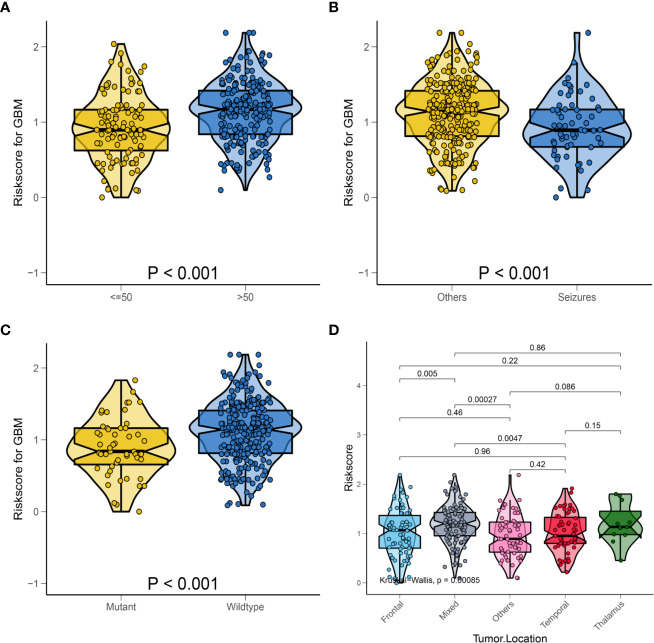
Relationship between HRS and some clinical characteristics in the whole data. **(A)** Box violin plot of HRS values of patients in age <= 50 years old group and age > 50 years old group; **(B)** Box violin plot of HRS values of patients in seizures group and others group; **(C)** Box violin plot of HRS values of patients in IDH mutant group and IDH wild-type group; **(D)** Box violin plot of HRS values of patients with tumor in frontal group, mixed group, others group, temporal group and thalamus group.

For better clinical application of the risk signature, we analyzed the HRS according to the age, first presenting symptoms, IDH mutation status, and tumor location in the overall database. As shown in **Figure 5A**, violin plots showed that patients aged >50 years had significantly higher HRSs than those of patients aged ≤50 years (P < 0.001). Furthermore, patients with seizures as the first presenting symptom had lower HRSs compared with those of patients with other symptoms (P < 0.001; **Figure 5B**). Similarly, we observed that patients with IDH-wild-type GBM had significantly higher HRSs than those of patients with IDH-mutant GBM (P < 0.001; **Figure 5C**). Regarding the tumor location, there was a significant difference in the HRS among tumor locations, especially in mixed areas (P < 0.001; **Figure 5D**).

**Figure 6:**
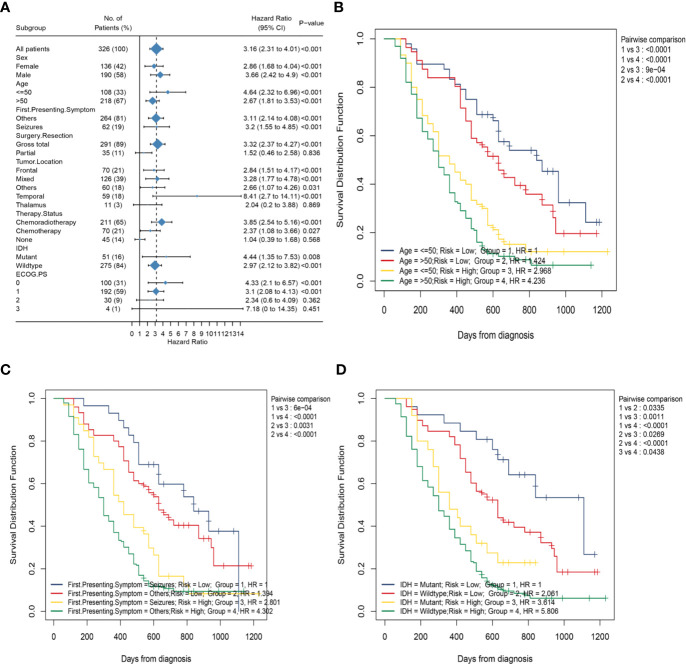
Subgroup analysis based on clinical characteristics in the whole data. **(A)** Forest plots of the associations between HRS and overall survival in various subgroups; **(B)** Kaplan–Meier curves of overall survival for patients in subgroups stratified by both HRS groups and age groups; **(C)** Kaplan–Meier curves of overall survival for patients in subgroups stratified by both HRS groups and first presenting symptom; **(D)** Kaplan–Meier curves of overall survival for patients in subgroups stratified by both HRS groups and IDH mutation status.

**Figure 7:**
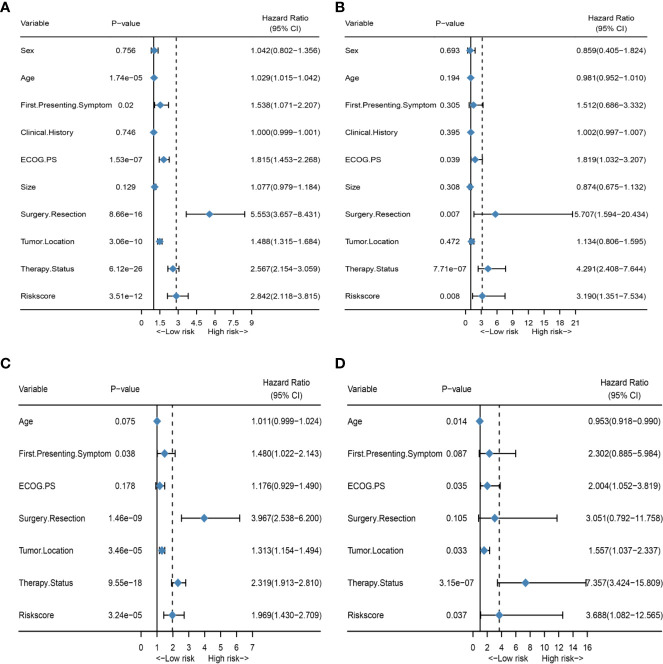
Subgroup analysis of the IDH mutant and wild-type populations. **(A)** Forest plot of univariate Cox regression analysis of all clinical covariates in the IDH wild-type population; **(B)** Forest plot of univariate Cox regression analysis of all clinical covariates in the IDH mutant population; **(C)** Forest plot of multivariate Cox regression analysis of significant clinical covariates in the IDH wild-type population; **(D)** Forest plot of multivariate Cox regression analysis of significant clinical covariates in the IDH mutant population.

To better explore the clinical significance of the HRS, we compared it individually with other clinical risk factors for subgroup analysis. As shown in **Figure 6A**, all patients were categorized into HRS high- or low-risk groups according to all significant subgroups, and the analysis showed that the high-risk group had significantly higher hazard ratios than those in the low-risk group, except among the “surgical resection-partial” (P = 0.84), “tumor location-thalamus” (P = 0.87), “ECOG PS-3 or 4” (P = 0.36 vs. P = 0.45) and “therapy status-none” (P = 0.57) subgroups. Considering the particularity of IDH mutant populations, we performed further subgroup analysis of the IDH mutant and wild-type populations respectively. In **Figures 7A–D**, after adjusting for variables such as the age, ECOG PS score, first presenting symptoms, tumor location, surgical resection, and therapy status, the results indicated that HRS was an independent prognostic factor, regardless of an IDH-mutant or IDH-wild-type status. These results indicated that the HRS can be widely applied to the risk prediction of most GBMs.

Subsequently, to investigate the association between the HRS and several clinical characteristics, we first developed the K-M curve to present the OS corresponding to the age, first presenting symptoms, and IDH mutation status subgroups between the HRS low-risk and high-risk groups. As shown in **Figure 6C**, the HRS high-risk group of GBM, regardless of whether or not seizures was the first presenting symptom, showed a significantly poorer OS than that in the HRS low-risk group (P < 0.001). However, the survival difference between patients in the seizures subgroup and patients in the other symptoms subgroup was not statistically significant for patients with GBM in the HRS low-risk or high-risk groups (P > 0.05). Similarly, patients in the HRS high-risk group had a significantly poorer OS than that in the low-risk group, regardless of age >50 years or age ≤50 years (P < 0.001; **Figure 6B**). There was no significant difference in survival between patients aged >50 years and patients aged ≤50 years in the HRS low-risk or HRS high-risk groups (P > 0.05). In addition, the OS of patients in the high-risk group was significantly lower than that of patients in the low-risk group, regardless of an IDH-mutant or IDH-wild-type status (P = 0.001 vs. P < 0.001; **Figure 6D**).Nevertheless, among patients in the HRS low-risk or HRS high-risk groups, the prognostic differences between the IDH-mutant subgroup and IDH-wild-type subgroup were statistically significant (P = 0.03 vs. P = 0.04).

To explore the predictive value of HRS in MGMT methylated population, we conducted a separate analysis of standard chemotherapy patients who had MGMT methylation analysis. In our results, the hazard ratio of patients with MGMT unmethylation was approximately twice that of patients with MGMT methylation. Based on the results of univariate and multivariate analyses, HRS proved to be an independent prognostic factor for OS (**Supplementary Figures S1A, B**). To develop a clinically relevant quantitative method for predicting the probability of patient mortality, we constructed a nomogram (**Supplementary Figure S1D**) integrating both HRS, MGMT status and clinical prognostic factors. The C-index of the nomogram was 0.83, and the calibration plots (**Supplementary Figure S1C**) showing the predicted probability of OS against the actual observed rates at 0.5 and 1 year were produced, suggesting good stability. These results implied that the HRS may be a comprehensive reflection of peripheral blood and helpful in identifying high-risk patients with GBM.

## Discussion

In recent decades, despite continuous improvements in neurosurgery techniques and innovations in new treatment methods, such as immunotherapy and molecular-targeted therapy, the survival time of patients with GBM is still not optimistic (4, 29, 30). Accumulating studies have demonstrated that peripheral blood test parameters play a remarkable role in the prognosis of various malignant tumors, such as lung cancer, gastric cancer, colorectal cancer, and hepatocellular carcinoma, as well as GBM (12–15, 31–33). Unlike genetic biomarkers, preoperative hematological markers can be easily obtained from routine blood tests, which are noninvasive and more cost-effective. A recent scoring system developed by He et al. based on the combination of plasma FIB and albumin levels can predict progression-free survival and OS in patients with high-grade gliomas (34). Another prognostic score simultaneously considering C-reactive protein and albumin levels is an effective prognostic tool for patients with GBM treated with radiotherapy and temozolomide (35). However, these scoring systems do not fully utilize the existing hematological markers that have been proven to have prognostic value, such as the NLR, PLR, PNI, RDW, and DD. Therefore, we summarized the available hematological markers with prognostic significance to establish a scoring system that could reflect inflammation, nutrition, and coagulation statuses simultaneously. In this study, we first adjusted 10 hematological markers to binary variables according to their optimal cutoff values, performed univariate Cox regression analysis on them in the training group, and further applied LASSO Cox regression to screen the optimal combination of hematological markers, that is, the HRPSS. The robustness of the HRPSS was then validated in the external validation group. The AUCs of the HRS in the training and validation groups were 0.78 and 0.79 at 2 years, respectively, indicating that the HRS has high predictive ability for long-term survival. Meanwhile, the HRS was corroborated as an independent prognostic factor *via* univariate and multivariate Cox analyses. To further improve the accuracy of the prediction of patients’ OS, we established a nomogram based on the HRS in the training set. The C-index of this nomogram was 0.81, while the C-index of the nomogram was 0.82 in the external validation set, which showed excellent predictive power. Interestingly, among the prognostic factors before surgery, the HRS was the dominant factor in the nomogram, indicating that the HRS might serve as a powerful preoperative prognostic factor.

Seizures as presenting symptoms associated with high‐grade gliomas account for approximately 30%–62% of patients, which is less than that associated with low‐grade gliomas (36). Presentation with seizures has traditionally been identified as a positive prognostic factor. This may be because seizures may trigger earlier presentation for care, thus accelerating diagnosis and initiating earlier treatment of smaller GBM (37). Mutations in *IDH* genes (*IDH*1 and *IDH*2) in GBM have been shown to predict better survival due to mutations in the nicotinamide adenine dinucleotide phosphate-dependent IDH encoded by it. Young age may be related to favorable genetic changes, such as *IDH* mutations and *ATRX* loss, which are associated with better clinical outcomes in patients with GBM (38, 39). A recent publication by Dietterle et al. revealed that the hazard ratio of unilobar tumors is twice that of multilobar tumors (40). Similarly, through analysis of 326 patients with GBM, we observed that patients aged >50 years, with non-seizures symptoms, with IDH wild-type status, and with tumors in mixed areas tended to have inferior OS than that of patients in other subgroups, which was consistent with the findings of previous studies. In addition, we also found that patients aged >50 years, with non-seizure symptoms, with IDH wild-type status, and with tumors in mixed areas had significantly higher HRSs. Today, in the clinic, clinical features such as the age, occurrence of seizures, IDH mutation status and MGMT methylation status remain important guidelines for risk stratification of patients and subsequent development of specific treatment plans. However, patients with the same stratification often have completely different prognoses despite being administered the same treatments. Obviously, factors other than clinical characteristics need to be considered to identify high-risk patients more accurately. Our results showed that patients in the HRS high-risk group had significantly poorer OS than that in the HRS-low group, regardless of whether or not they had seizures. In the HRS low-risk group, there was no significant difference in survival between the seizures group and other symptoms group. Similar conclusions were reached in the analysis of age and IDH mutation status. Therefore, the HRS is expected to serve as a powerful supplement to clinical features, to identify high-risk patients more accurately and further develop more individualized therapy for high-risk patients with GBM.

Epigenetic silencing of the MGMT gene by promoter methylation has been associated with longer overall survival in patients with glioblastoma who received alkylating chemotherapy with TMZ. The MGMT methylation status may lead to its subsequent failure to protect tumors from cytotoxic damage induced by TMZ, thereby predicting the benefits of TMZ chemotherapy (6, 41). Among 69 standard chemotherapy patients who received MGMT methylation analysis, HRS was still an independent prognostic factor. The hazard ratio of patients with MGMT unmethylation was approximately twice that of patients with MGMT methylation, which indicated that MGMT methylation is a powerful protective factor for the OS of GBMs. The C-index of the nomogram including MGMT status and HRS was 0.83, which showed a high predictive ability. However, due to the relatively low economic level of Henan Province, the sample size was small. We believe that this result should be interpreted with caution.

Recently, studies have demonstrated that hematological markers may reflect the tumor microenvironment to some extent in GBM (10, 17, 19, 20). Inflammation in the tumor microenvironment promotes angiogenesis, tumor invasion, and metastasis, while increasing tumor cell proliferation and enhancing their survival, and also destroying the body’s innate and adaptive immune responses. Neutrophils and monocytes are usually regarded as potent immune suppressors in the tumor microenvironment (10, 42). These cells can remodel the extracellular matrix and promote angiogenesis, which may stimulate tumor cell migration and metastasis (43). Derived from circulating monocytes, tumor-associated macrophages may support tumor progression and angiogenesis through the secretion of growth factors and cytokines (44). In contrast, tumor-infiltrating lymphocytes are considered important in anti-cancer immune responses by producing cytokines and inducing cytotoxic cell death (45). Platelets can mediate tumor cell growth, angiogenesis, and proliferation by releasing vascular endothelial growth factor, hepatocyte growth factor, basic fibroblast growth factor, and angiopoietin-1, as well as other angiogenesis and tumor growth factors. Platelets have a clear role in protecting tumor cells from immune elimination and supporting tumor metastasis (46). In addition, platelets can activate several signaling pathways in cancer cells, leading to a more invasive mesenchymal-like phenotype (47). In accordance with the critical role of neutrophils, lymphocytes, and monocytes in tumor progression, high NLR, PLR, and MLR suggest a rather poor prognosis for patients with cancer. In the present study, the weighting coefficients of the preoperative NLR, PLR, and MLR calculated through LASSO Cox regression analysis were 0.30, 0.10, and 0.36, respectively, indicating that higher values of the NLR, PLR, and MLR are correlated with unfavorable clinical outcomes in patients, which is consistent with the findings of previous studies (13–15).

GBM is associated with preoperative hypercoagulability and a high risk of venous thromboembolism (VTE). There is a close link between the mechanism by which tumors arise and systems that govern blood coagulation from the early stages of the disease. The coagulation system is an important aspect of the unique vascular microenvironment in which tumors proliferate and progress. The induction of a state of systemic hypercoagulability by a tumor-expressing substrate has been explained by the discovery of circulating microparticles that originate from tumor antigens or tissue factors and derived from the membranes of leukocytes, platelets, endothelial cells, and tumor cells following activation or apoptosis. Disorders in the coagulation–fibrinolysis system in patients with GBM, such as low levels of FIB and DD, lead to hypercoagulability in the body, which stimulates the formation of VTE and increases the risk of surgical treatment (17, 48). Simultaneously, previous studies have shown that a hypercoagulable state correlates with poor clinical outcomes in patients with GBM (16, 49). In our study, the weighting coefficients of FIB and DD levels used in the calculation formula of the HRPSS were 0.24 and 0.27, showing that high levels of FIB and DD are associated with adverse prognosis.

In addition, another retrospective analysis revealed that a low PNI, which reflects the nutritional and immunological statuses, is significantly associated with short OS in patients with GBM (23). It has been previously investigated with regard to GBM prognosis; for example, Lally et al. pointed to low HBG levels, which are associated with anemia, as an adverse prognostic factor (21). A recent publication by Liang et al. revealed that a high RDW in patients with glioma may be attributed to a variety of underlying metabolic abnormalities, such as inflammation, oxidative stress, and poor nutritional status, which are negatively correlated with OS in patients with GBM (26). Increased LDH levels are considered an important indicator of increased glycolysis and have been confirmed to play an important role in tumor metabolism, development, invasion, and patient prognosis (50, 51). Correspondingly, patients with preoperative hyperglycemia may have tumors with more malignant features due to sustained exposure to GLC, which is the favored energy substrate of cancer cells (18, 52). In our results, the coefficients of the PNI, RDW, LDH level, and GLC level used to construct the HRPSS were -0.23, 0.44, 0.32, and 0.15, respectively, showing that high RDW, LDH level, and GLC level and low PNI are correlated with poor prognosis, which is in line with previously published findings. These indicators may, respectively, have certain guiding significance for the prognosis of GBM in certain respects, which provides a basis for the construction of a scoring system used before surgery.

There were some limitations to our study. First, this was a single-center retrospective study that may have led to bias in selection and analysis. Second, the scoring system has low predictive ability for patients with short-term survival, such as patients who have undergone partial resection, with tumors in the thalamus, who have not received adjuvant therapy, and who have poorer performance status. Therefore, caution should be exercised when applying the HRPSS to these patients. Third, since the values of the hematological markers were entered into LASSO Cox regression as categorical variables, the optimal cutoff value needs to be further verified in future studies. However, we are the first, to our knowledge, to develop a hematological prognostic scoring system using LASSO Cox regression analysis. Moreover, compared to previous studies, we incorporated all significant indicators to build an HRPSS, which compensated for the limitation that traditional single markers cannot comprehensively reflect the microenvironment of the patient’s body, such as coagulation function, inflammation, and nutritional status. According to the ROC curves, the predictive ability of the HRS was better than that of any single indicator used to construct it, which showed its significant predictive potential. Considering the limited number of studies previously conducted, further studies are needed to explore the important prognostic value of hematological markers in GBM.

In summary, we constructed and validated a novel HRPSS in two different datasets, which was demonstrated to be an independent prognostic factor for patients with GBM. Moreover, we also established a nomogram, which integrated both the HRS derived from this HRPSS, as well as clinical factors, to quantitatively predict the OS of patients with GBM. In addition, we explored the relationship between the HRS and clinical characteristics. These findings should be useful to clinicians and patients with GBM regarding treatment decisions, management, and prognosis.

## Data Availability Statement

The original contributions presented in the study are included in the article/**Supplementary Material**, further inquiries can be directed to the corresponding author.

## Author Contributions

CZ collected and analyzed the data and wrote the paper. L-QL assisted in collecting the data and participated in the writing. F-DY, R-LW, M-KW, D-XS, X-YG, and WD assisted in the design of this study. X-TW is responsible for the integrity of the data and the accuracy of the data analysis. All authors contributed to the article and approved the submitted version.

## Funding

This research was supported by the Key Research Projects of Henan Higher Education (No.19A320075).

## Conflict of Interest

The authors declare that the research was conducted in the absence of any commercial or financial relationships that could be construed as a potential conflict of interest.

## Supplementary Material

Supplementary Figure 1 Analysis of standard chemotherapy patients undergoing MGMT methylation analysis. **(A)** Forest plot of univariate Cox regression analysis of all clinical covariates; **(B)** Forest plot of multivariate Cox regression analysis of significant clinical covariates; **(C)** Calibration chart to verify the accuracy of the nomogram; **(D)** Nomogram to predict the probability of patient mortality based on HRS, MGMT status and other clinical characteristics.
