# Supplementary figures and images for: A Hematological-Related Prognostic Scoring System for Patients With Newly Diagnosed Glioblastoma
Source: Front Oncol. 2020 Dec 10;10:591352. doi: 10.3389/fonc.2020.591352 (PMC7758450; doi:10.3389/fonc.2020.591352)

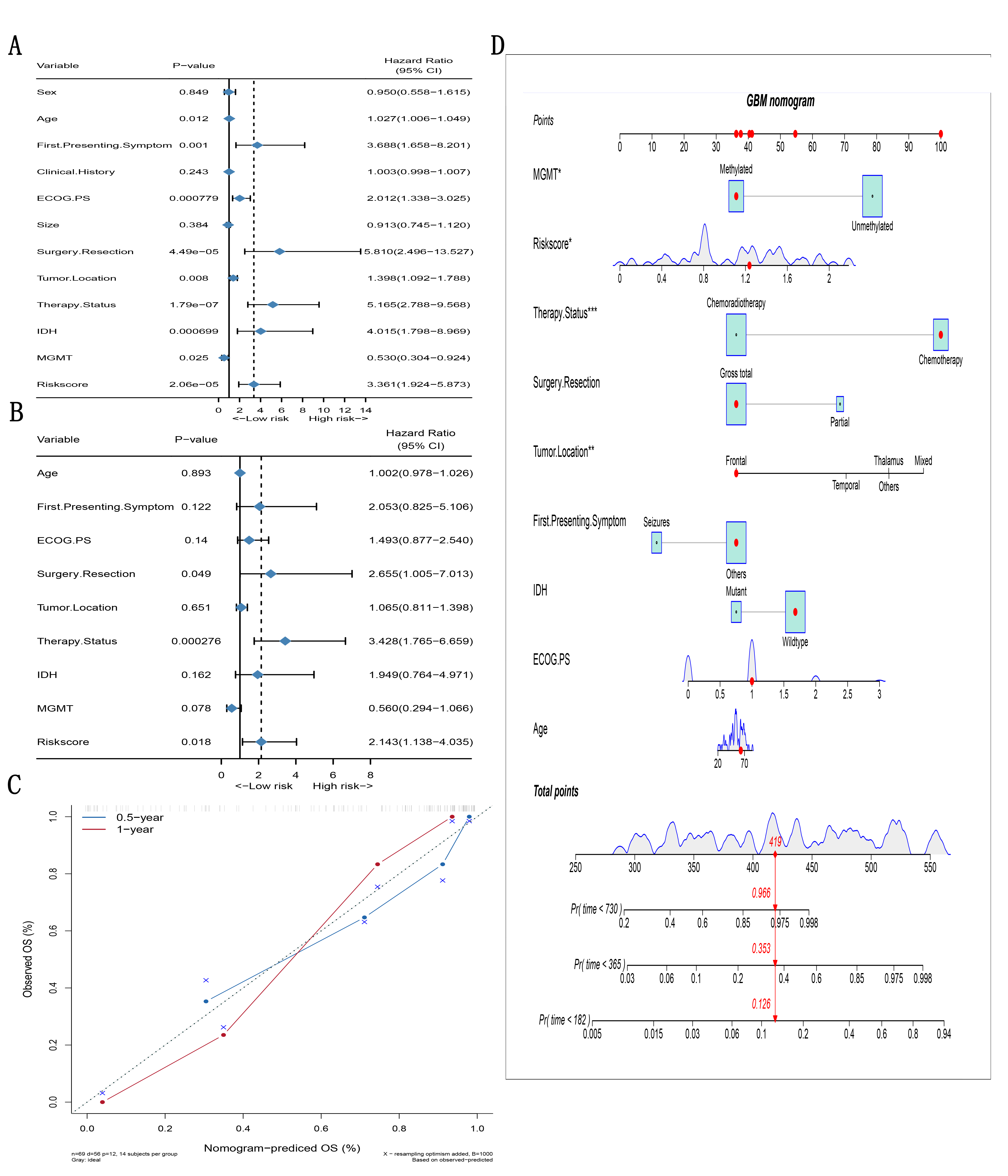

Supplement: Supplementary Figure 1 — Analysis of standard chemotherapy patients undergoing MGMT methylation analysis. (A) Forest plot of univariate Cox regression analysis of all clinical covariates; (B) Forest plot of multivariate Cox regression analysis of significant clinical covariates; (C) Calibration chart to verify the accuracy of the nomogram; (D) Nomogram to predict the probability of patient mortality based on HRS, MGMT status and other clinical characteristics. [file Image_1.tif]
