# Supplementary material for: A Hematological-Related Prognostic Scoring System for Patients With Newly Diagnosed Glioblastoma
Source: Front Oncol. 2020 Dec 10;10:591352. doi: 10.3389/fonc.2020.591352 (PMC7758450; doi:10.3389/fonc.2020.591352)
Supplement: Supplementary file 2 [file Table_1.docx]

**Supplementary Table S1.** The optimal cut-off values and weighting coefficients of covariates used to construct the HRPSS.

| **covariates** | **cutoff values** | **coefficients** |
| --- | --- | --- |
| RDW | 12.8 | 0.44 |
| HBG | 114 | * |
| GLC | 5.4 | 0.15 |
| LDH | 179 | 0.32 |
| FIB | 3.3 | 0.24 |
| DD | 0.15 | 0.27 |
| NLR | 2.3 | 0.30 |
| PLR | 97.7 | 0.10 |
| MLR | 0.59 | 0.36 |
| PNI | 54.8 | -0.23 |

# *RDW, red blood cell distribution width; HBG, hemoglobin; GLC, glucose; LDH, lactate dehydrogenase; FIB, fibrinogen; DD, D-dimer; NLR, the neutrophil-to-lymphocyte ratio; PLR, the platelet-to-lymphocyte ratio; MLR, the monocyte-to-lymphocyte ratio; PNI, the prognostic nutrition index.*

# Supplementary Table S2. Clinical characteristics of 69 patients undergoing MGMT methylation analysis.

| **Characteristic** | **MGMT- methylated (n=25, 36.2%)** | **MGMT- unmethylated (n=44, 63.8%)** |  |
| --- | --- | --- | --- |
| **Sex** | | | |
| Male | 18(72.0%) | 20(45.5%) |  |
| Female | 7(28.0%) | 24(54.5%) |  |
| **Age, years** | | | |
| Mean | 54.8 | 56.2 |  |
| Range | 21-75 | 23-85 |  |
| **First Presenting Symptom** | | | |
| Seizures | 6(24.0%) | 8(18.2%) |  |
| Others | 19(76.0%) | 36(81.8%) |  |
| **Clinical History, days** | | | |
| Median | 15 | 27.5 |  |
| Range | 2-180 | 1-365 |  |
| **ECOG PS** | | | |
| 0  1  2 | 12(48.0%)  12(48.0%)  1(4.0%) | 12(27.3%)  27(61.4%)  4(9.1%) |  |
| 3 | 0(0%) | 1(2.3%) |  |
| **Size, cm** | | | |
| <=5 | 13(52.0%) | 30(68.2%) |  |
| >5 | 12(48.0%) | 14(31.8%) |  |
| **Surgery Resection** | | | |
| Gross Total | 36(81.8%) | 25(100.0%) |  |
| Partial | 8(18.2%) | 0(0%) |  |
| **Tumor Location** | | | |
| Frontal | 4(16.0%) | 14(31.8%) |  |
| Temporal | 6(24.0%) | 7(15.9%) |  |
| Mixed | 8(32.0%) | 16(36.4%) |  |
| Thalamus | 1(4.0%) | 4(9.1%) |  |
| Others | 6(24.0%) | 3(6.8%) |  |
| **Therapy Status** | | | |
| Chemoradiotherapy | 20(80.0%) | 24(54.5%) |  |
| Chemotherapy | 5(20.0%) | 20(45.5%) |  |
| **IDH** | | | |
| Mutant | 7(28.0%) | 7(15.9%) |  |
| Wildtype | 18(72.0%) | 37(84.1%) |  |
| **HRS** | | | |
| High | 12(48.0%) | 28 (63.6%) |  |
| Low | 13(52.0%) | 16(36.4%) |  |
| **OS, months** |  |  |  |
| Mean | 19.4 | 11.4 |  |
| Range | 3-39.5 | 2-41 |  |
| **Survival Status** |  |  |  |
| Alive | 5(20.0%) | 8(18.2%) |  |
| Dead | 20(80.0%) | 36(81.8%) |  |

*ECOG PS, the Eastern Cooperative Oncology Group performance status score; IDH, isocitrate dehydrogenase-1/2 mutations; MGMT,* *O6-methylguanine-DNA methyltransferase; HRS, the hematological risk score; OS, overall survival;* [Chemoradiotherapy](http://dict.youdao.com/w/chemoradiotherapy/#keyfrom=E2Ctranslation)*: radiotherapy plus concomitant and adjuvant temozolomide; Chemotherapy: only adjuvant chemotherapy with temozolomide; None: without any postoperative adjuvant treatment.*
